# Diagnostic performance of biomarkers for differentiating active tuberculosis from latent tuberculosis: a systematic review and Bayesian network meta-analysis

**DOI:** 10.3389/fmicb.2024.1506127

**Published:** 2024-12-20

**Authors:** Ji Hun Jeong, Sung Ryul Shim, Sangah Han, Inhwan Hwang, Chunhwa Ihm

**Affiliations:** ^1^Department of Laboratory Medicine, Daejeon Eulji Medical Center, Eulji University, Daejeon, Republic of Korea; ^2^Department of Biomedical Informatics, College of Medicine, Konyang University, Daejeon, Republic of Korea; ^3^Konyang Medical Data Research Group KYMERA, Konyang University Hospital, Daejeon, Republic of Korea; ^4^Department of Blood Management Services, and Daejeon Eulji Medical Center, Eulji University, Daejeon, Republic of Korea; ^5^Department of Hematooncology, Daejeon Eulji Medical Center, Eulji University, Daejeon, Republic of Korea

**Keywords:** tuberculosis, latent tuberculosis, biomarkers, differential diagnosis, interleukin-2

## Abstract

**Background:**

PCR and culture tests are used together to confirm the diagnosis of active tuberculosis (TB). Due to the long culture period, if the PCR test is negative, it takes a significant amount of time for the culture result to be available. Interferon-*γ* release assays (IGRAs), which are widely used to diagnose TB or latent tuberculosis infection (LTBI), cannot effectively discriminate TB from LTBI. The purpose of this study is to analyze the diagnostic performance of various markers for differentiating between TB from LTBI.

**Methods:**

PubMed-Medline, EMBASE, Cochrane Library, and Web of Science were searched up to the end of May 2024, without restrictions on publication date and population. Articles describing the diagnostic value of at least one biomarker for differentiating between TB and LTBI were included. The QUADAS-2 tool was used to assess study quality. Two independent researchers assessed the articles using Preferred Reporting Items for Systematic Reviews and Meta-Analyses (PRISMA) guidelines. The network meta-analysis (NMA) was performed for diagnostic tools of 11 groups used to differentiate TB from LTBI.

**Results:**

Out of 164 identified articles, 159 reports were included in the systematic review and 58 in the meta-analysis. Seventy results from 58 reports accounting for 9,291 participants were included. When measuring interleukin-2 (IL-2) after stimulation with latency antigen, the most significant odds ratio was shown in terms of sensitivity, specificity, positive predictive value and negative predictive value. The values were 9.46, 18.5, 11.30, and 9.61, respectively.

**Conclusion:**

This study shows that the IL-2 level after stimulation with latent antigen is a potential biomarker for differentiating TB from LTBI.

**Systematic review registration:**

https://www.crd.york.ac.uk/prospero/display_record.php?ID=CRD42024542996.

## 1 Introduction

Tuberculosis (TB) remains a significant public health problem and a leading cause of infectious death. TB was the world’s second leading cause of death from a single infectious pathogen, after coronavirus disease (COVID-19) (World Health Organization, 2023). TB is caused by the bacillus *Mycobacterium tuberculosis*, which is spread when symptomatic infected people expel bacteria into the air by coughing. The spectrum of the disease ranges from asymptomatic and non-transmissible latent TB infection (LTBI) to highly active, transmissible TB disease. After infection with *M. tuberculosis*, approximately 5% of healthy adults will develop active TB within 2 years (Menzies et al., 2018). Individuals with LTBI face an ongoing risk of developing active TB through reactivation based on the host immune response.

Diagnosing TB involves a detailed medical history, clinical examination, and radiological, microbiological, immunological, molecular-biological, and histological investigations, where available (Acharya et al., 2020). However, the clinical presentation of TB is diverse; definite diagnosis can be challenging due to the limited sensitivity of the nucleic acid amplification test and *M. tuberculosis* cultures, especially in extrapulmonary forms of TB. The TB PCR and culture are sputum-based tests, causing problems for patients who cannot produce sufficient sputum, often seen in children and patients with extrapulmonary disease.

Moreover, there is no gold standard test for LTBI. Because of low bacterial burden, the diagnosis of LTBI is indirect and depends on evidence of a cellular immune response to mycobacterial antigens. The most commonly used tests for LTBI diagnosis are the intradermal tuberculin test (TST) and interferon (IFN)-*γ* release assays (IGRAs) (Zellweger et al., 2020). False positives in TST occur mainly in patients who have had the BCG vaccine, have infections with non-tuberculosis mycobacteria (NTM), or are immunosuppressed, such as those with AIDS (Acharya et al., 2020). False negatives can occur in patients with recent TB infection, ancient TB infection, recent live virus vaccination, and some viral infections (measles and chicken pox) (Zellweger et al., 2020). Incorrect methods or interpretation of results, especially in young children, can also lead to false negatives (Zellweger et al., 2020).

Alternatives to TST, such as IGRA, are diagnostic tests based on the detection the *in vitro* secretion of IFN-*γ* by lymphocytes stimulated with peptides specifically encoded by *M. tuberculosis*. Two tests are widely used (the QuantiFERON-TB Plus and the T-SPOT.TB test), which differ in the laboratory procedure but rely on the same principle. IGRAs provide an accurate diagnosis of *M. tuberculosis* infection, but do not differentiate between TB and LTBI (Carranza et al., 2020; Zellweger et al., 2020). Although TST and IGRA can diagnose LTBI, these methods can only differentiate infected individuals from healthy person and cannot distinguishing TB from LTBI. Because the treatments for TB and LTBI are different, misdiagnosis between TB and LTBI leads to the undertreatment of TB patients and overtreatment of LTBI patients. Moreover, there are differences in contagiousness, discrimination between TB and LTBI is very important in TB control. Many researches recently showed the immune response against a wide range of stage-specific antigens and evaluated the concentrations of biomarkers after stimulation with various antigens (Meier et al., 2018). Among the *M. tuberculosis* antigens, the antigens that are highly expressed in the latent tuberculosis state and that can differentiate LTBI from active TB were reported as latency-associated antigens (Meier et al., 2018; Bai et al., 2016; Chegou et al., 2012). However, there is currently no diagnostic test that can clearly distinguish between TB and LTBI.

This study aims to analyze potential biomarkers for differentiating TB from LTBI and confirm the performance of various modalities for discriminating between TB and LTBI through a network meta-analysis (NMA) of published direct comparison studies.

## 2 Materials and methods

### 2.1 Data sources and search strategy

A search strategy using PubMed, EMBASE and the Cochrane Library was developed in collaboration with two independent authors (SR Shim and JH Jeong) up to the end of May 2024. We also manually searched the reference lists of identified publications for additional studies. We used the controlled terminology of Medical Subject Headings (MeSH) for PubMed and Cochrane, and Emtree for EMBASE, along with text keywords to find studies related to the diagnostic markers in TB and LTBI (Supplementary Table S1). This study was registered in the PROSPERO database (registration number: CRD42024542996) and conducted following the Preferred Reporting Items for Systematic Reviews and Meta-Analyses (PRISMA) statement (Hutton et al., 2015).

### 2.2 Study selection

The inclusion criteria for the NMA were as follows: (1) Studies where TB was recognized based on clinical symptoms and a positive result from at least one form of microbiological evidence, such as staining of acid-fast bacilli (AFB), AFB culture, or a molecular tests. LTBI cases were defined as apparently healthy individuals who had a history of close contact with TB patients and displayed positive results of TST or IGRA but showed no signs or symptoms of TB disease and had negative cultures for *M. tuberculosis*. (2) The antigens that are highly expressed in the latent tuberculosis state and that can differentiate LTBI from active TB were classified as latency-associated antigens in this study. Index tests and reference standards including the use of IFNg_TB_Ag (IFN-*γ* detection after TB antigen stimulation), CD4_T cell (CD4 T-cell detection without stimulation), CD8_T cell (CD8 T-cell detection without stimulation), IFNg_LatencyAg (IFN-γ detection after latency antigen stimulation), IL10_TB_Ag (interleukin-10 detection after TB antigen stimulation), IL13_TB_Ag (interleukin-13 detection after TB antigen stimulation), IL2_LatencyAg (interleukin-2 detection after latency antigen stimulation), IL2_TB_Ag (interleukin-2 detection after TB antigen stimulation), IL5_TB_Ag (interleukin-5 detection after TB antigen stimulation), IP10_TB_Ag (IP-10 detection after TB antigen stimulation), and TNFɑ_TB_Ag (TNF-*α* detection after TB antigen stimulation) for differentiating TB and LTBI. (3) Outcomes including differential diagnostic performance such as sensitivity, specificity, positive predictive value (PPV) and negative predictive value (NPV).

Studies were not published in original articles such as letters, conference abstracts, and case reports were excluded. We primarily retrieved English publications. Two investigators independently determined the eligibility of the obtained literature.

### 2.3 Data extraction and quality assessment

For this systematic review and NMA, two investigators (SR Shim and JH Jeong) independently extracted data from selected articles, resolving disagreements by discussion and consensus. The extracted data included the first author, published time, country, TB incidence rate, number of TB patients and LTBI subjects, sensitivity, specificity, PPV and NPV. Each study was analyzed to retrieve the diagnostic performance of various markers for differentiating TB from LTBI based on the reference standard. Only studies providing such complete information were included in the NMA.

According to the Quality Assessment of Diagnostic Accuracy Studies tool-2 (QUADAS-2) recommended by the Cochrane Collaboration (Whiting et al., 2011), two investigators independently reviewed the quality of the articles. The QUADAS-2 evaluated the risk of bias and applicability of eligible studies across four domains: patient selection, index test, reference standard, and flow and timing. Selection bias exists in participants. In the index test part, whether the participants were detected in blind ways is critical. Information and disease progression bias are related to the reference standard. Signaling questions were included to help judge the quality of eligible articles. Disagreements were resolved by consensus.

### 2.4 Statistical analysis

The NMA was performed for different biomarkers and tools used to differentiate LTBI from TB. The various diagnostic tools were classified into 11 groups, and then NMA was performed for the different categories.

For Bayesian NMA, specific graphical analysis was completed using the “gemtc” package in R software v.4.3.1 (R Foundation for Statistical Computing) (Shim et al., 2019). The simulation was conducted by putting the prior distribution and probability into the Markov Chain Monte Carlo (MCMC). After that, the optimal convergence model was selected by reviewing the trace plot, normal distribution plot, and the MCMC standard error of the generated posterior distribution. Through this, the posterior probability of the effect sizes of each biomarker could be calculated in summary statistics (sensitivity, specificity, PPV, and NPV). A consistency test between direct and indirect comparisons was performed through node-splitting assessments.

In the Bayesian approach, the optimal probability of individual treatments being selected can be obtained using the generated posterior distribution, which represents a kind of priority between treatments as a Surface Under the Cumulative Ranking Curve (SUCRA); the larger the SUCRA value, the higher the rank of the intervention (Shim et al., 2019; Rücker and Schwarzer, 2015; Salanti et al., 2011). The analysis pooled the odds ratios (ORs) and 95% credible intervals (CrI). A two-sided *p*-value of ≤0.05, or not containing a null value (OR = 1) within the 95% CrIs, was considered statistically significant.

In addition, diagnostic test accuracy (DTA) was also conducted to specifically confirm the characteristics of individual biomarkers according to various covariates. The results showed the pooled estimation between summary statistics (sensitivity, specificity, PPV, NPV, and diagnostic OR) and 95% confidential interval (CI) for differentiating TB from LTBI. We used the bivariate random-effects model for analysis and pooling of the diagnostic performance measures across studies, as well as comparisons between different index tests (Reitsma et al., 2005; Shim and Lee, 2019). We also used the model to create hierarchical summary receiver operating characteristic curves (SROC) and to estimate the area under the curve (AUC) (Rutter and Gatsonis, 2001).

## 3 Results

### 3.1 Study selection and characteristics

**Figure 1:**
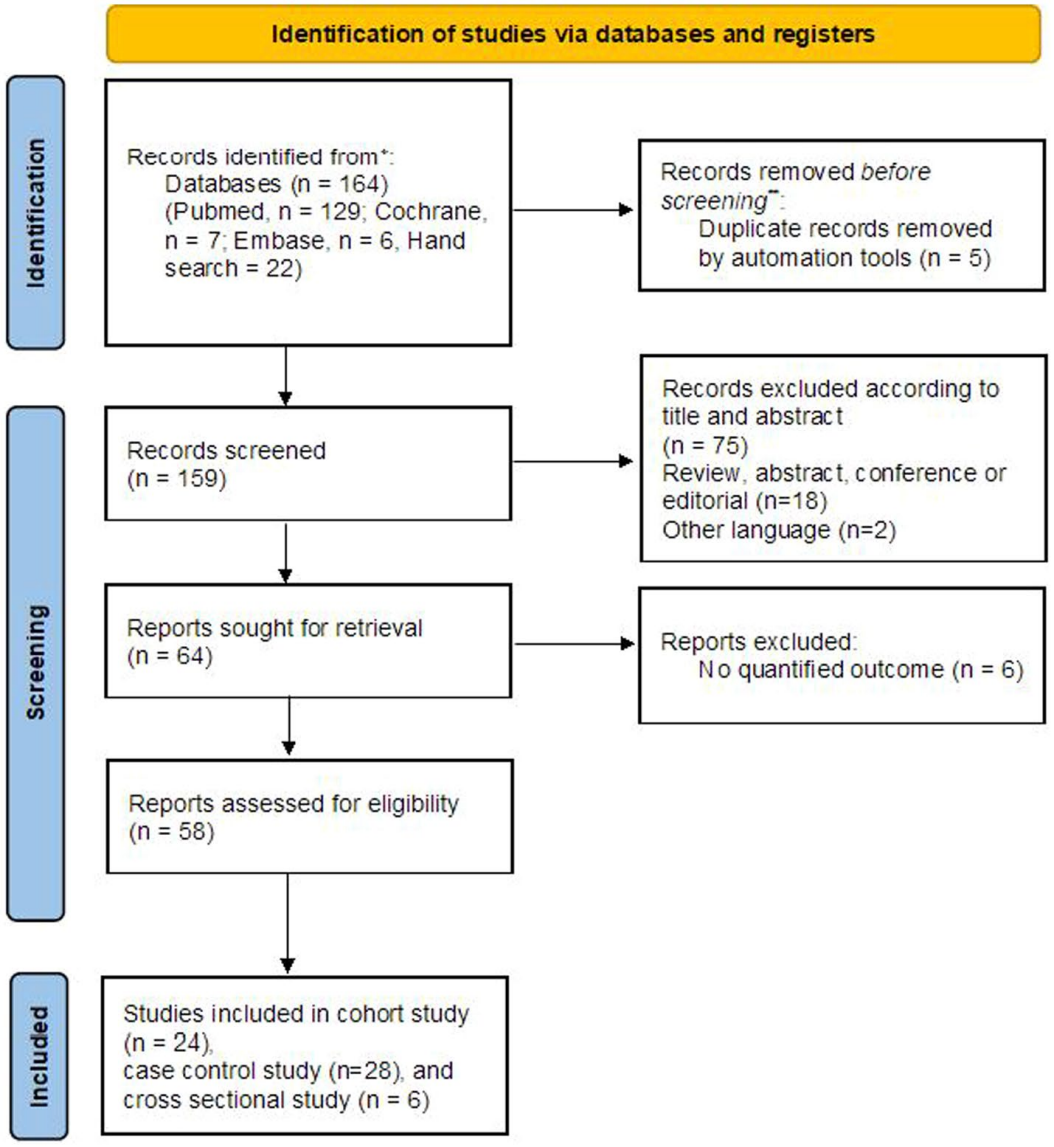
Flow chart of the identified and included articles.

A total of 164 literature citations were retrieved from three independent databases. After removing 5 duplicates, we read titles and abstracts and excluded 95 records (75 were irrelevant topics, 18 were reviews, abstracts or letters, and two were not written in English). Ultimately, a total of 9,291 patients from 58 articles were included (Bai et al., 2016; Balcells et al., 2018; Baumann et al., 2014; Bayaa et al., 2021; Cao et al., 2018; Chegou et al., 2013; Chiappini et al., 2012; Corrìa et al., 2019; Delemarre et al., 2021; Della Bella et al., 2018; El-Sheikh et al., 2021; Gourgouillon et al., 2012; Grassi et al., 2021; Jeong et al., 2015; Kaewseekhao et al., 2020; Kamakia et al., 2017; Katakura et al., 2020; Kim et al., 2015; Li, 2016; Li et al., 2023; Li et al., 2022; Luo et al., 2019; Luo et al., 2020; Luo et al., 2021c; Luo et al., 2021a; Luo et al., 2021e; Luo et al., 2021f; Luo et al., 2021b; Luo et al., 2021d; Luo et al., 2022; Luo et al., 2023; Mamishi et al., 2016; Mamishi et al., 2019; Masungi et al., 2002; Molicotti et al., 2011; Molicotti et al., 2015; Movahedi et al., 2017; Nonghanphithak et al., 2017; Peng et al., 2020; Petrone et al., 2018; Sali et al., 2018; Sandhu et al., 2012; Sun et al., 2016; Sutherland et al., 2010; Suzukawa et al., 2016; Tang et al., 2020; Temmerman et al., 2004; Wang et al., 2012; Wang et al., 2013; Wang et al., 2019; Wang et al., 2021; Wen et al., 2017; Won et al., 2017; Wu et al., 2017; Yang et al., 2015; Yao et al., 2017; Zhang et al., 2022; Zhou et al., 2017), and the details of the study screening process are shown in Figure 1. The 58 studies were mainly performed in five countries: China (48.3%), Italy (12.1%), Iran (5.2%), Japan (5.2%) and Korea (5.2%). The study subjects were mainly from areas with moderate to high TB burden (72.4%), diagnostic tests using stimulators (75.9%), and assay detecting cytokines or chemokines (51.7%), and immunoassays (62.1%). All selected studies were prospective case–control, cohort, and cross sectional studies. The detailed characteristics of all the studies are shown in Supplementary Table S2.

### 3.2 Quality assessment

**Figure 2:**
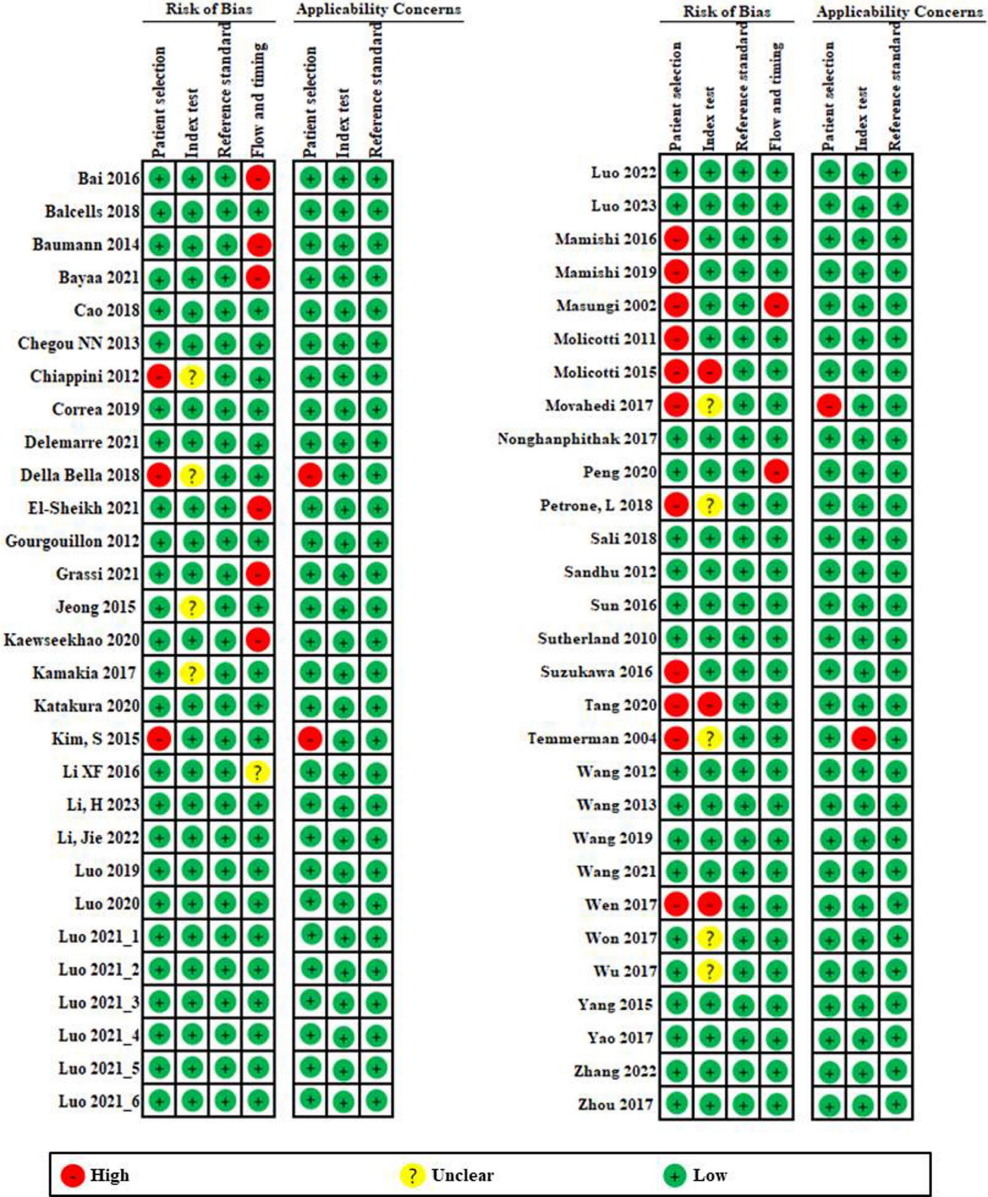
Risk of bias and applicability concerns graph based on 15-item modified Quality Assessment of Diagnostic Accuracy Studies. The overall quality of the included studies was deemed to be satisfactory.

A summary of the risk of bias and applicability concerns based on 15-item QUADAS-2 is presented in Figure 2. First, looking at the risk of bias, patient selection (Chiappini et al., 2012; Della Bella et al., 2018; Kim et al., 2015; Mamishi et al., 2016; Mamishi et al., 2019; Masungi et al., 2002; Molicotti et al., 2011; Molicotti et al., 2015; Movahedi et al., 2017; Nonghanphithak et al., 2017; Petrone et al., 2018; Suzukawa et al., 2016; Tang et al., 2020; Temmerman et al., 2004; Wen et al., 2017) and flow and timing (Bai et al., 2016; Baumann et al., 2014; Bayaa et al., 2021; El-Sheikh et al., 2021; Grassi et al., 2021; Kaewseekhao et al., 2020; Masungi et al., 2002; Peng et al., 2020) showed relatively high risk compared to other domains. Although the index test was clearly established, six studies were graded as high risk due to lack of explanation. Reference tests showed that all studies were low risk.

In applicability concerns, patient selection and index testing showed low risk in all but three studies (Della Bella et al., 2018; Kim et al., 2015; Movahedi et al., 2017) and one study (Temmerman et al., 2004). Reference tests showed that all studies were low risk. Overall, quality of the included studies had a low risk of bias and an acceptable level of applicability.

### 3.3 Diagnostic test accuracy of detection markers for discrimination of TB and LTBI

The diagnostic performance results of tools according to disease groups and TB burden are presented in Supplementary Table S3. The sensitivity ranged from 0.746 to 0.895; the specificity ranged from 0.822 to 0.901; the PPV ranged from 0.844 to 0.909; and the NPV ranged from 0.721 to 0.906 (Supplementary Table S3). The pooled sensitivity, pooled specificity, pooled PPV, and pooled NPV of the diagnostic methods used in this analysis were all above 0.85, showing good diagnostic performance. In the TB group, the results were slightly higher than the LTBI group.

**Figure 3:**
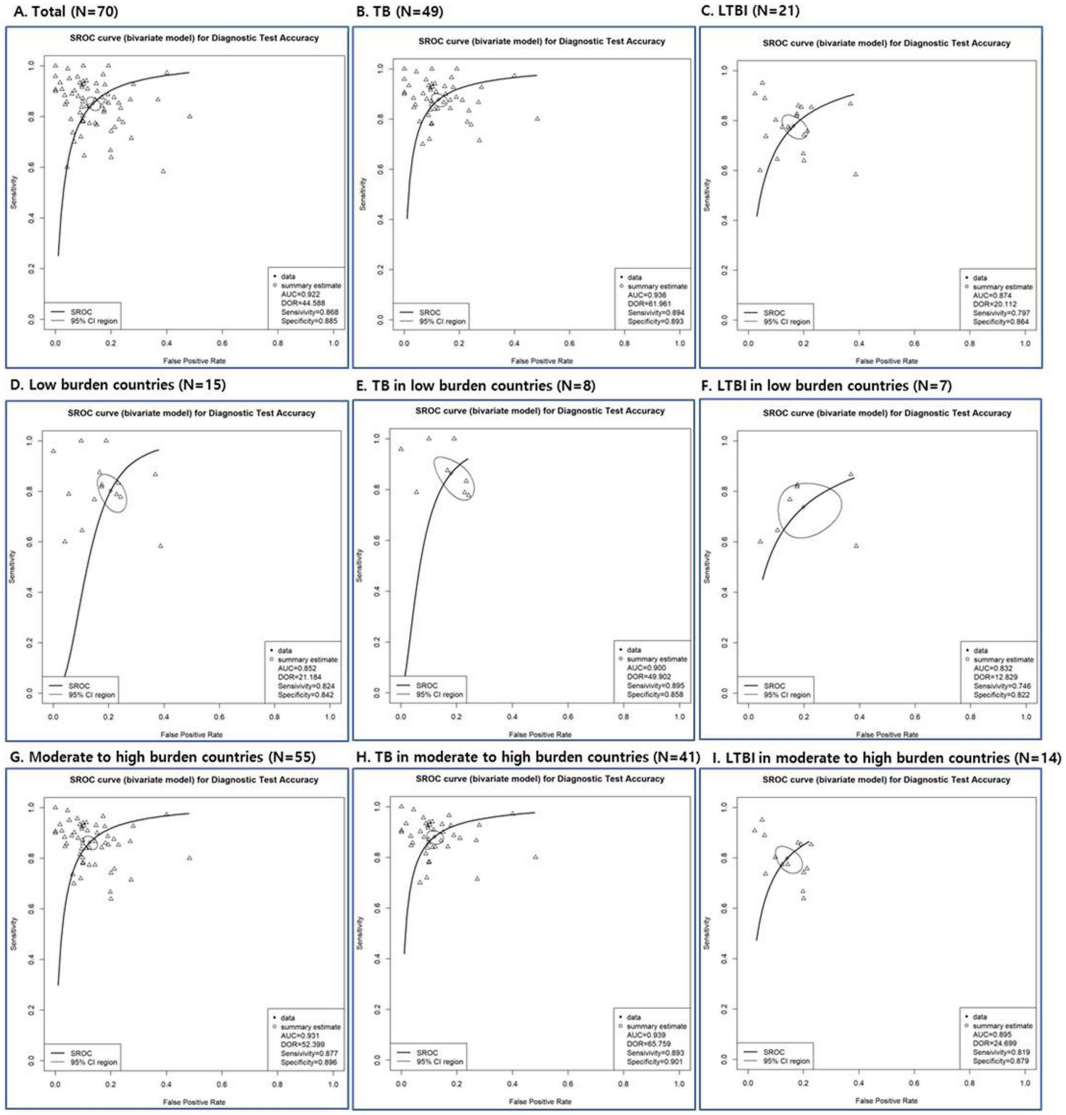
Summary receiver operating characteristic (SROC) curves of diagnostic tests depending on total studies **(A)**, disease status (TB: **B, E, H** and LTBI: **C, F, I**) and TB burden (low burden country: **D, E, F** and moderate to high burden country: **G, H, I**).

According to SROC curve, AUC was 0.832 to 0.939 (Figure 3; Supplementary Table S4). The AUC of SROC analysis for total was 0.922 (sensitivity 0.852, specificity 0.865); for TB, it was 0.936 (sensitivity 0.878, specificity 0.875); for LTBI, it was 0.874 (sensitivity 0.778, specificity 0.837) (Figure 3; Supplementary Table S4). The diagnostic tools used in the study had high overall diagnostic performance, but among them, the overall diagnostic performance was best in the TB disease group.

### 3.4 Network meta-analysis (NMA)

**Figure 4:**
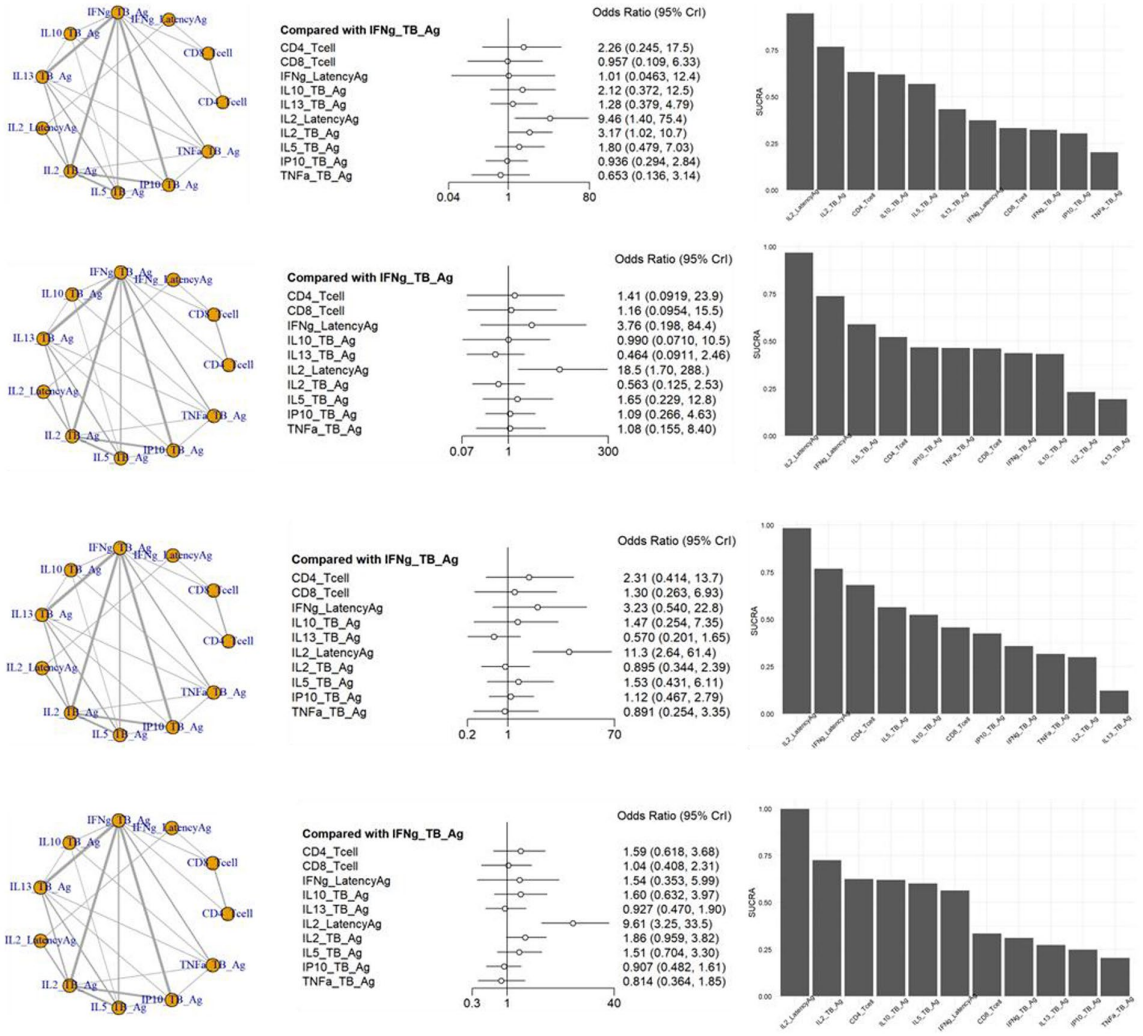
Schematic diagram of the network of evidence used in network meta-analysis, forest plots and surface under the cumulative ranking area (SCURA) in **(A)** sensitivity, **(B)** specificity, **(C)** positive predictive value (PPV) and **(D)** negative predictive value (NPV) of various markers for differentiating TB from LTBI.

The inconsistency tests for NMA assumption were analyzed using the node-splitting approach, and the findings (*p* > 0.05 for all) indicate consistency across the direct and indirect comparisons for all outcomes. In the NMA shown in Figure 4, a variety of visualization techniques were used to elucidate the comparative diagnostic performance of various markers for TB from LTBI. The network plots provide a comprehensive illustration of all the direct and indirect treatment comparisons, establishing a visual network of the evidence base. Adjacent to these plots, network forest plots present the effect sizes along with their 95% credible intervals for each comparison, offering a detailed statistical evaluation of the diagnostic performance (Supplementary Table S5). Complementing these, the SUCRA bar charts distill the cumulative data into a ranked probability format, demonstrating the likelihood of each diagnostic test being most efficacious. A network plot of the 66 included results is depicted in Figure 4. There were many results compared with methods of measuring IFNg_TB_Ag, and a total of 11 methods could be compared. As a result of sensitivity, the odds ratio of IL2_LatencyAg and IL2_TB_Ag were 9.46 [95% CrI: 1.40, 75.40] and 3.17 [95% CrI: 1.02, 10.07], respectively, which were statistically significantly high. The SUCRA also showed the rankings of IL2_LatencyAg, IL2_TB_Ag, and CD4_Tcell. TNFa showed the lowest ranking, which was lower than the IFNg_TB_Ag currently used in IGRA.

In the specificity analysis, only IL2_LatencyAg showed a statistically significant high value of 18.50 [95% CrI: 1.70, 288.00]. The SUCRA result also showed the rankings of IL2_LatencyAg, IFNg_LatencyAg, and IL5_TB_Ag. IL13_TB_Ag showed the lowest ranking.

The PPV analysis results showed that only IL2_LatencyAg had a statistically significant high value of 11.30 [95% CrI: 2.64, 61.40]. In the SUCRA results, IL2_LatencyAg ranked highest, followed by IFNg_LatencyAg and CD4_Tcell. IL13_TB Ag showed the lowest ranking.

In the NPV analysis results, only IL2_LatencyAg showed a statistically significant high value of 9.61 [95% CrI: 3.25, 33.50]. IL2_LatencyAg also ranked highest, while TNFa showed the lowest ranking in the SUCRA.

### 3.5 Publication bias

**Figure 5:**
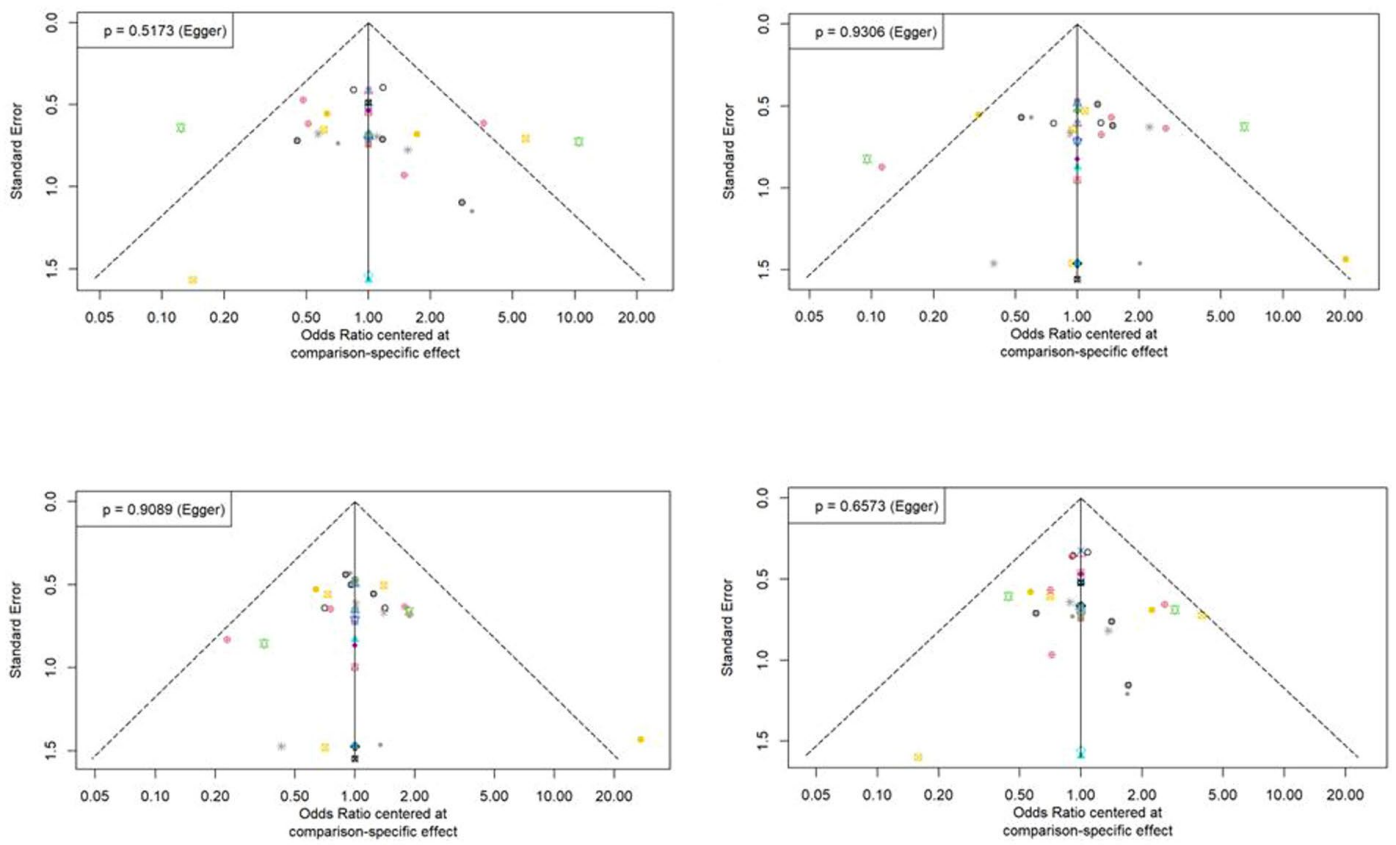
Funnel plot asymmetry test for assessing the discrimination power between tuberculosis and latent tuberculosis. Non-significant slope indicates that no significant bias was found.

The statistical approaches to publication bias in 58 studies using funnel plots are shown in Figure 5. Individual studies were distributed symmetrically about the combined effect size and toward the top of the graph. Additionally, the Egger’s regression test, which mathematically analyzed publication bias, also suggested that there was no evidence of publication bias or small-study effect in this NMA (all *p* > 0.05).

## 4 Discussion

This systematic review and NMA included 58 studies encompassing 9,291 patients, summarizing the evidence on diagnostic accuracy outcomes (sensitivity, specificity, PPV, and NPV) for discrimination between TB and LTBI. A total of 11 methods could be compared. Forest plots shows that the odds ratio of IL2_LatencyAg is statistically significant in sensitivity, specificity, PPV and NPV, and the values were 9.46 [95% CrI: 1.40, 75.40], 18.5 [95% CrI: 1.70, 288.00], 11.30 [95% CrI: 2.64, 61.40], and 9.61 [95% CrI: 3.25, 33.50], respectively. The SUCRA values of the performance of 11 different diagnostic methods for the discrimination between TB and LTBI indicated that “IL2_LatencyAg” ranked first based on sensitivity, specificity, PPV, and NPV. There are many other test methods that show superiority over the IFNg_TB_Ag used as a commercial kit, but TNF a and IL13_TB Ag were ranked low in performance. Among the diagnostic tools assessed, IL2_LatencyAg emerged as the most effective, ranking highest in sensitivity, specificity, PPV, and NPV. This method’s diagnostic odds ratios were significantly higher than other methods, underscoring its potential as a preferred diagnostic tool for clinical practice.

TB is an infectious disease spread through the respiratory tract, while LTBI has no symptoms and not contagious but carries a risk of progressing to TB depending on the individual’s immune status (Menzies et al., 2018). Since there are differences in treatment for each condition, distinguishing between TB and LTBI is essential to reduce the burden on people with TB. However, no laboratory tool is currently available for differential diagnosis. Immunoassay-based methods could be clinically useful tools for distinguishing TB from LTBI due to their speed and cost-effectiveness. Immunological results can be obtained more quickly than microbial culture and at a lower cost than molecular or imaging tests. Therefore, developing immunologic methods to distinguish TB from LTBI and improving the diagnostic performance of existing tests can play a positive role in reducing the prevalence of tuberculosis and controlling the infection.

IGRAs, immune-based blood tests that measure IFN-*γ* (T-cell responses to TB-specific antigens) are widely used for diagnosing LTBI. Commercial IGRAs are based on quantifying IFN-γ after brief lymphocyte stimulation (16–24 h) that allows detection of effector and effector memory T cells (Lanzavecchia and Sallusto, 2000). To improve diagnostic performance, enhancements are being made to the antigens stimulated *in vitro*. Besides early secreted antigenic target 6 (ESAT-6) and culture filtrate protein 10 (CFP-10), the new generation of IGRA includes the new TB-specific secreted protein TB 7.7 as antigen that boosts the host cellular immune response, thereby increasing sensitivity for identifying LTBI (Armand et al., 2014). The reagent used to measure only IFN-γ produced by CD4 T-cells has also been used recently to measure IFN-γ produced by CD8 T-cells (Jomehpour et al., 2023). However, detecting only IFN-γ cannot differentiate TB from LTBI (Diel et al., 2011). Besides IFN-γ, interleukin-2 (IL-2) is an additional cytokine produced by helper T cells, stimulating both helper T cells and cytotoxic T lymphocytes (Sharma et al., 2014). It has been reported that the IL-2 levels differ between TB and LTBI (Biselli et al., 2010; Borgstrøm et al., 2012; Carranza et al., 2020). It has been also reported that IL-2/IFN-γ is an useful value for differentiating TB from LTBI (Biselli et al., 2010). Our evidence generated by NMA can provide important decision support that IL2_LatencyAg is an effective tool for discrimination of TB and LTBI, which can be added to commercial kits to enhance differential diagnostic performance.

In addition to IL2_LatencyAg, NMA results confirmed various test methods with superior performance compared to the current IGRA method (IFNg_TB_Ag). This showed the possibility of improving performance by combining other test targets with the current test method. In particular, it showed the potential for improved sensitivity by additionally measuring IL-2 with the currently used IGRA (IFNg_TB_Ag). It is thought that adding IL-2 as a measurement substance to the currently used IGRA method will be relatively easy to commercialize.

Various targets or immunological markers have been proposed over the last decade for the differential diagnosis between TB and LTBI. We want to explore whether cytokines, chemokine or other detection tools besides IFN-γ could improve the ability to distinguish between different TB infection statuses. Our study found that detecting IL-2 after stimulation using latency antigen or TB antigen are good candidates, potentially increasing the diagnostic potential of current methods for discriminating TB and LTBI.

While our study provides valuable insights, there are some limitations. The heterogeneity in study designs and patient populations may affect the generalizability of our findings. Each detection method is diverse, such as ELISA, ELISPOT, Luminex, RT-PCR, and flow cytometry, the detection target, and stimulator differ. There are differences in whether or not a stimulator is used, and when using a stimulator, there are various combinations such as using a single antigen as a TB antigen or using multiple antigens together. However, it was difficult to compare all combinations one-to-one, so during subgroup analysis, they were grouped into similar categories and analyzed for comparison. The relatively small sample size may have also influenced the result of the analysis due to low numbers of individuals in IL-2 category. The NMA results included the results of 273 patients from only three studies (Della Bella et al., 2018; Movahedi et al., 2017; Chiappini et al., 2012). The test method used in the study was IL-2 based ELISpot, which is inconvenient in terms of testing, making large-scale studies difficult to conduct in reality. It is necessary to derive results using larger-scale studies through the development of a commercially available simple ELISA-based technology and evaluation of a sufficient number of cases.

When performing immunoassays using Luminex or ELISA, the results may not be accurate when measuring low-concentration substances, and there may be significant differences between the testing methods (Platchek et al., 2020). Fortunately, most of the biomarkers we used in NMA were high-concentration (Suzukawa et al., 2016), but when screening new low-concentration markers and comparing different testing methods, it is necessary to develop a precise testing method that can provide consistent results and unify the testing methods.

## 5 Conclusion

In conclusion, our systematic review and meta-analysis demonstrate that the IL-2 detection after latency antigen stimulation has the potential to serve as biomarker for discrimination between TB and LTBI. In addition to the method of measuring IL-2 after stimulation with latency antigen, the sensitivity of measuring IL-2 after stimulation with TB antigen was statistically significantly higher than the currently used IGRA method. There are limitations of heterogeneity in the types of testing methods and biomarkers, so it is thought that the performance of differential diagnosis can be improved if IL-2 is added to the current testing method rather than being used as a single marker for differential diagnosis. In addition, large-scale performance evaluations are needed for clinical utility of IL-2 alone or in combination with other biomarkers.

## Data Availability

The original contributions presented in the study are included in the article/Supplementary material, further inquiries can be directed to the corresponding author.
