# Supplementary material for: Diagnostic performance of biomarkers for differentiating active tuberculosis from latent tuberculosis: a systematic review and Bayesian network meta-analysis
Source: Front Microbiol. 2024 Dec 20;15:1506127. doi: 10.3389/fmicb.2024.1506127 (PMC11695403; doi:10.3389/fmicb.2024.1506127)
Supplement: Supplementary file 1 [file Table_1.docx]

**TABLE S1 Search queries**

| **PubMed and Cochrane Library** | |
| --- | --- |
|  | 1. Disease  ("Latent Tuberculosis"[Mesh] OR "Tuberculosis Infection, Latent"[tiab] OR "Latent Tuberculosis Infection"[tiab]) AND ("Tuberculosis"[Mesh] OR "Tuberculosis Infection"[tiab] OR "Active tuberculosis"[tiab]) |
|  | 2. Intervention  ("Interferon-gamma Release Tests"[Mesh] OR "Interferon gamma Release Tests"[tiab] OR "Interferon-gamma Release Assays"[tiab] OR "Interferon gamma Release Assays" [tiab] OR "IGRA"[tiab] OR "Tuberculin Test"[Mesh] OR "Intradermal Tests"[Mesh] OR "Tuberculin Test"[tiab] OR "Intradermal Tests"[tiab] OR "TST"[tiab] OR "tuberculin skin test"[tiab] OR “PPD”[tiab] OR "QuantiFERON"[tiab] OR "QFT-Plus"[tiab] OR "QFT-TB"[tiab] OR “enzyme-linked immunospot” OR “ELISpot” OR "T-SPOT"[tiab] OR "TB-SPOT"[tiab] OR "TB-IGRA"[tiab] OR "heparin-binding hemagglutinin adhesin"[tiab] OR "heparin-binding hemagglutinin"[tiab] OR "HBHA"[tiab]) AND ("Antigens"[Mesh] OR "antigen"[tiab] OR "Biomarkers"[Mesh] OR "biomarker"[tiab] OR "Cytokines"[Mesh] OR "Cytokine"[tiab])  3. Outcome  ("Diagnosis, Differential"[Mesh] OR "Differential Diagnosis"[tiab] OR "Differential Diagnoses"[tiab] OR "latent and active"[tiab] OR "discrimination"[tiab] OR "distinguish"[tiab]) AND ("Area Under Curve"[Mesh] OR "Sensitivity and Specificity"[Mesh]) |
|  |  |
| **Embase** | |
|  | 1. Disease  ('tuberculosis'/exp OR 'tuberculosis':ti,ab) AND ('latent':ti,ab AND ‘tuberculosis':ti,ab) |
|  | 2. Intervention  (‘interferon-gamma release tests’/exp OR 'quantiferon'/exp OR'elispot'/exp OR't-spot'/exp OR ‘mycobacterium tuberculosis test kit’:ti,ab)  3. Outcome  ('discrimination'/exp OR 'discrimination':ti,ab) AND ('distinguish'/exp OR 'distinguish':ti,ab) |

**TABLE S2 Main characteristics of studies with 9291 patients included in the meta-analysis**

| **Author** | **Year** | **Country** | **TB burden** | **Study design** | **Population** | **Stimulation** | **Stimulator** | **Marker** | **Detection method** | **Total** | **TP** | **FP** | **FN** | **TN** |
| --- | --- | --- | --- | --- | --- | --- | --- | --- | --- | --- | --- | --- | --- | --- |
| Bai | 2016 | China | moderate to high | case control | adolescents, adults | stimulation | LA Ag | cytokines/ chemokines | immunoassay | 167 | 93 | 5 | 23 | 46 |
| Balcells | 2018 | Chille | moderate to high | cohort | adolescents, adults | stimulation | TB Ag | cytokines/ chemokines | immunoassay | 56 | 29 | 5 | 5 | 17 |
| Baumann | 2014 | Germany | low | case control | adolescents, adults | stimulation | TB Ag | antibody | immunoassay | 89 | 35 | 11 | 7 | 36 |
| Bayaa | 2021 | Madagascar | moderate to high | cohort | adolescents, adults | unstimulation |  | transcriptomics | molecular test | 70 | 40 | 3 | 4 | 23 |
| Cao | 2018 | China | moderate to high | case control | adults | unstimulation |  | antibody | others | 40 | 19 | 1 | 1 | 19 |
| Chegou NN | 2013 | South Africa | moderate to high | cohort | children | unstimulation |  | cytokines/ chemokines | immunoassay | 41 | 13 | 7 | 2 | 19 |
| Chiappini | 2012 | Italy | low | case control | children | stimulation | TB Ag | cytokines/ chemokines | immunoassay | 46 | 25 | 4 | 0 | 17 |
| Correa | 2019 | Brazil | moderate to high | case control | adults | unstimulation |  | others | immuno-phenotyping | 29 | 13 | 2 | 2 | 12 |
| Delemarre | 2021 | Netherlands | low | cohort | adults | unstimulation |  | cytokines/ chemokines | immunoassay | 40 | 20 | 2 | 0 | 18 |
| Della Bella | 2018 | Italy | low | case control | adults | stimulation | TB Ag | cytokines/ chemokines | immunoassay | 161 | 70 | 0 | 3 | 88 |
| El-Sheikh | 2021 | Egypt | low | case control | children | unstimulation |  | cytokines/ chemokines | molecular test | 51 | 14 | 8 | 4 | 25 |
| Gourgouillon | 2012 | France | low | case control | children | stimulation | TB Ag | cytokines/ chemokines | immunoassay | 14 | 7 | 1 | 1 | 5 |
| Grassi | 2021 | Italy | low | case control | adults | unstimulation |  | cell proportion | immuno-phenotyping | 37 | 15 | 1 | 4 | 17 |
| Jeong | 2015 | Korea | moderate to high | case control | adults | stimulation | TB Ag | cytokines/ chemokines | immunoassay | 53 | 31 | 2 | 2 | 18 |
| Kaewseekhao | 2020 | Thailand | moderate to high | case control | adults | unstimulation |  | protein pattern | others | 46 | 26 | 0 | 0 | 20 |
| Kamakia | 2017 | Japan | low | case control | adults | stimulation | TB Ag | cytokines/ chemokines | immunoassay | 35 | 14 | 1 | 5 | 15 |
| Katakura | 2020 | Japan | low | cross section | adults | stimulation | TB Ag | combination | combination | 340 | 123 | 42 | 33 | 142 |
| Kim, S | 2015 | Korea | moderate to high | case control | adults | stimulation | TB Ag | cytokines/ chemokines | molecular test | 50 | 20 | 6 | 8 | 16 |
| Li XF | 2016 | China | moderate to high | case control | unclear | stimulation | TB Ag | cytokines/ chemokines | immunoassay | 129 | 61 | 2 | 11 | 55 |
| Li, H | 2023 | China | moderate to high | cohort | adults | unstimulation |  | cytokines/ chemokines | immunoassay | 148 | 87 | 0 | 9 | 52 |
| Li, Jie | 2022 | China | moderate to high | cohort | adolescents, adults | unstimulation |  | others | others | 120 | 56 | 1 | 4 | 59 |
| Luo | 2019 | China | moderate to high | cohort | adults | unstimulation |  | combination | immunoassay | 99 | 44 | 4 | 6 | 45 |
| Luo | 2020 | China | moderate to high | cohort | adults | stimulation | TB Ag | combination | immunoassay | 119 | 61 | 5 | 5 | 48 |
| Luo | 2021_1 | China | moderate to high | cohort | adults | stimulation | TB Ag | immuno-phenotype | immuno-phenotyping | 64 | 28 | 6 | 1 | 29 |
| Luo | 2021_2 | China | moderate to high | cohort | adults | stimulation | TB Ag | combination | combination | 2059 | 856 | 94 | 241 | 868 |
| Luo | 2021_3 | China | moderate to high | cohort | adults | stimulation | TB Ag | combination | combination | 116 | 50 | 11 | 4 | 51 |
| Luo | 2021_4 | China | moderate to high | cohort | adults | stimulation | TB Ag | combination | combination | 709 | 272 | 39 | 40 | 358 |
| Luo | 2021_5 | China | moderate to high | cohort | adults | stimulation | TB Ag, lymphocyte stimulator | combination | combination | 113 | 41 | 6 | 8 | 58 |
| Luo | 2021_6 | China | moderate to high | cohort | adults | stimulation | TB Ag, lymphocyte stimulator | immuno-phenotype | immuno-  phenotyping | 111 | 44 | 5 | 10 | 52 |
| Luo | 2022 | China | moderate to high | cohort | adults | stimulation | TB Ag | combination | combination | 669 | 343 | 14 | 4 | 308 |
| Luo | 2023 | China | moderate to high | cohort | adults | stimulation | TB Ag | combination | combination | 1246 | 542 | 60 | 90 | 554 |
| Mamishi | 2016 | Iran | moderate to high | case control | adults | stimulation | TB Ag | cytokines/ chemokines | immunoassay | 61 | 23 | 6 | 8 | 24 |
| Mamishi | 2019 | Iran | moderate to high | case control | adults | stimulation | TB Ag | cytokines/ chemokines | immunoassay | 59 | 24 | 14 | 6 | 15 |
| Masungi | 2002 | Belgium | low | case control | unclear | stimulation | LA Ag | cytokines/ chemokines | immunoassay | 49 | 15 | 1 | 10 | 23 |
| Molicotti | 2011 | Italy | low | case control | unclear | stimulation | LA Ag | cytokines/ chemokines | immunoassay | 63 | 19 | 7 | 4 | 33 |
| Molicotti | 2015 | Italy | low | case control | children, adults | stimulation | LA Ag | cytokines/ chemokines | immunoassay | 83 | 43 | 4 | 13 | 23 |
| Movahedi | 2017 | Iran | moderate to high | case control | adults | stimulation | TB Ag | cytokines/ chemokines | immunoassay | 66 | 25 | 7 | 8 | 26 |
| Nonghanphithak | 2017 | Thailand | moderate to high | case control | adults | unstimulation |  | cytokines/ chemokines | immunoassay | 86 | 42 | 8 | 6 | 30 |
| Peng | 2020 | China | moderate to high | case control | adults | stimulation | TB Ag | antibody | others | 160 | 90 | 9 | 10 | 51 |
| Petrone, L | 2018 | Italy | low | case control | adults | stimulation | TB Ag | cytokines/ chemokines | immunoassay | 67 | 21 | 12 | 15 | 19 |
| Sali | 2018 | Italy | low | cohort | children | stimulation | LA Ag | cytokines/ chemokines | immunoassay | 64 | 39 | 7 | 6 | 12 |
| Sandhu | 2012 | Peru | moderate to high | case control | adolescents, adults | unstimulation |  | others | others | 204 | 134 | 10 | 17 | 43 |
| Sun | 2016 | China | moderate to high | case control | adults | stimulation | TB Ag | combination | immunoassay | 108 | 59 | 1 | 6 | 42 |
| Sutherland | 2010 | Gambia | moderate to high | cohort | adults | stimulation | TB Ag | combination | immunoassay | 56 | 35 | 8 | 1 | 12 |
| Suzukawa | 2016 | Japan | low | case control | adults | stimulation | TB Ag | cytokines/ chemokines | immunoassay | 60 | 20 | 3 | 11 | 26 |
| Tang | 2020 | China | moderate to high | case control | adults | stimulation | LA Ag | cytokines/ chemokines | immunoassay | 96 | 49 | 7 | 8 | 32 |
| Temmerman | 2004 | Belgium | low | case control | unclear | stimulation | LA Ag | cytokines/ chemokines | immunoassay | 101 | 45 | 8 | 10 | 38 |
| Wang | 2012 | China | moderate to high | cross section | adolescents, adults | stimulation | TB Ag | combination | immunoassay | 107 | 51 | 5 | 15 | 36 |
| Wang | 2013 | China | moderate to high | cross section | adolescents, adults | stimulation | TB Ag | cytokines/ chemokines | immunoassay | 47 | 18 | 2 | 7 | 20 |
| Wang | 2019 | China | moderate to high | cohort | adults | stimulation | TB Ag | combination | molecular test | 53 | 24 | 1 | 4 | 24 |
| Wang | 2021 | China | moderate to high | cross section | adolescents, adults | stimulation | TB Ag | cytokines/ chemokines | immunoassay | 62 | 35 | 4 | 6 | 17 |
| Wen | 2017 | China | moderate to high | cross section | adults | stimulation | LA Ag | combination | immunoassay | 101 | 10 | 17 | 5 | 69 |
| Won | 2017 | Korea | moderate to high | cohort | adults | stimulation | TB Ag | cytokines/ chemokines | immunoassay | 51 | 23 | 3 | 13 | 12 |
| Wu | 2017 | China | moderate to high | cohort | adults | stimulation | TB Ag | cytokines/ chemokines | immunoassay | 61 | 21 | 4 | 4 | 32 |
| Yang | 2015 | China | moderate to high | cohort | adults | unstimulation |  | cytokines/ chemokines | immuno-  phenotyping | 129 | 24 | 6 | 3 | 96 |
| Yao | 2017 | China | moderate to high | cohort | adolescents, adults | stimulation | TB Ag | combination | immunoassay | 30 | 18 | 0 | 2 | 10 |
| Zhang | 2022 | China | moderate to high | cross section | adults | stimulation | TB Ag, LA Ag | combination | immunoassay | 93 | 48 | 6 | 9 | 30 |
| Zhou | 2017 | China | moderate to high | cohort | adults | stimulation | TB Ag | combination | immuno-  phenotyping | 107 | 66 | 3 | 3 | 35 |

Abbreviation: TB Ag, tuberculosis antigen; LA Ag, latency associated antigen; TP, true positive; FP, false positive; FN, false negative; TN, true negative

**TABLE S3 Subgroup analysis of the diagnostic tools for discrimination of the LTBI and active TB in univariate model.**

| **Covariate** | **Subgroup** | **Sensitivity [95% CI]** | **Specificity [95% CI]** | **PPV [95% CI]** | **NPV [95% CI]** | **DOR [95% CI]** |
| --- | --- | --- | --- | --- | --- | --- |
| **Diagnosis** | Total (N=70) | 0.868 [0.849, 0.888] | 0.885 [0.866, 0.903] | 0.891 [0.872, 0.910] | 0.851 [0.827, 0.875] | 44.588 [33.075, 60.109] |
|  | TB (N=49) | 0.894 [0.874, 0.914] | 0.893 [0.871, 0.914] | 0.900 [0.880, 0.920] | 0.886 [0.865, 0.906] | 61.961 [43.805, 87.643] |
|  | LTBI (N=21) | 0.797 [0.758, 0.837] | 0.864 [0.827, 0.900] | 0.861 [0.814, 0.908] | 0.761 [0.704, 0.819] | 20.112 [13.183, 30.681] |
| **Low burden country** | Total (N=15) | 0.824 [0.758, 0.890] | 0.842 [0.786, 0.898] | 0.848 [0.793, 0.902] | 0.820 [0.751, 0.889] | 21.184 [11.210; 40.034] |
|  | TB (N=8) | 0.895 [0.823, 0.968] | 0.858 [0.780, 0.935] | 0.852 [0.768, 0.936] | 0.906 [0.841, 0.971] | 49.902 [14.034, 177.444] |
|  | LTBI (N=7) | 0.746 [0.663; 0.829] | 0.822 [0.736, 0.909] | 0.844 [0.779, 0.910] | 0.721 [0.632, 0.810] | 12.830 [6.115, 26.919] |
| **Moderate to high burden country** | Total (N=55) | 0.877 [0.858, 0.896] | 0.896 [0.878; 0.914] | 0.902 [0.884; 0.920] | 0.858 [0.833, 0.883] | 52.399 [38.338, 71.618] |
|  | TB (N=44) | 0.893 [0.872; 0.913] | 0.901 [0.881; 0.921] | 0.909 [0.890, 0.927] | 0.882 [0.861; 0.903] | 65.760 [46.275, 93.449] |
|  | LTBI (N=14) | 0.819 [0.777, 0.861] | 0.879 [0.840, 0.919] | 0.867 [0.804, 0.931] | 0.780 [0.707, 0.854] | 24.699 [15.613, 39.074] |

Abbreviations: TB, tuberculosis; LTBI, latent tuberculosis infection; PPV, positive predictive values; NPV, negative predictive value; DOR, diagnostic odds ratio; CI, confidence interval.

**TABLE S4 Subgroup analysis of the diagnostic tools for discrimination of the LTBI and active TB in bivariate model.**

| **Covariate** | **Subgroup** | **AUC** | **Sensitivity [95% CI]** | **Specificity [95% CI]** |
| --- | --- | --- | --- | --- |
| **Diagnosis** | Total (N=70) | 0.922 | 0.852 [0.829, 0.872] | 0.865 [0.844, 0.884] |
|  | TB (N=49) | 0.936 | 0.878 [0.855, 0.897] | 0.875 [0.850, 0.897] |
|  | LTBI (N=21) | 0.874 | 0.778 [0.737, 0.814] | 0.837 [0.797, 0.871] |
| **Low burden country** | Total (N=15) | 0.852 | 0.803 [0.735, 0.856] | 0.793 [0.748, 0.832] |
|  | TB (N=8) | 0.900 | 0.864 [0.783, 0.918] | 0.820 [0.756, 0.871] |
|  | LTBI (N=7) | 0.832 | 0.737 [0.641, 0.815] | 0.803 [0.696, 0.879] |
| **Moderate to high burden country** | Total (N=55) | 0.931 | 0.863 [0.840, 0.883] | 0.876 [0.854, 0.895] |
|  | TB (N=44) | 0.939 | 0.881 [0.858, 0.901] | 0.881 [0.855, 0.903] |
|  | LTBI (N=14) | 0.895 | 0.798 [0.751, 0.837] | 0.859 [0.816, 0.894] |

Abbreviations: TB, tuberculosis; LTBI, latent tuberculosis infection; AUC, area under curve; CI, confidence interval

**TABLE S5 *P* values between markers in sensitivity, specificity, positive predictive value and negative predictive value**

**(A) Sensitivity**

|  | **IL2_Latency Ag** | **IL2_TB_ Ag** | **IFNg_Latency Ag** | **IFNg_TB_ Ag** | **IL13_TB_ Ag** | **IL5_TB_ Ag** | **IP10_TB_ Ag** | **TNFa_TB_ Ag** | **CD4_T cell** | **CD8_T cell** |
| --- | --- | --- | --- | --- | --- | --- | --- | --- | --- | --- |
| **IL2_LatencyAg** |  | 0.021 | 0.029 |  |  |  |  |  |  |  |
| **IL2_TB_Ag** |  |  |  | 0.284 | 0.917 | 0.429 | 0.649 | 0.115 |  |  |
| **IFNg_LatencyAg** |  |  |  |  |  |  |  |  |  | 0.031 |
| **IFNg_TB_Ag** |  |  |  |  |  | 0.461 | 0.214 |  | 0.050 | 0.028 |
| **IL13_TB_Ag** |  |  |  |  |  | 0.440 | 0.021 | 0.311 |  |  |
| **IL5_TB_Ag** |  |  |  |  |  |  | 0.819 |  |  |  |
| **IP10_TB_Ag** |  |  |  |  |  |  |  | 0.269 |  |  |

**(B) Specificity**

|  | **IL2_Latency Ag** | **IL2_TB_ Ag** | **IFNg_Latency Ag** | **IFNg_TB_ Ag** | **IL13_TB_ Ag** | **IL5_TB_ Ag** | **IP10_TB_ Ag** | **TNFa_TB_ Ag** | **CD4_T cell** | **CD8_T cell** |
| --- | --- | --- | --- | --- | --- | --- | --- | --- | --- | --- |
| **IL2_LatencyAg** |  | 0.665 | 0.666 |  |  |  |  |  |  |  |
| **IL2_TB_Ag** |  |  |  | 0.841 | 0.812 | 0.006 | 0.950 | 0.193 |  |  |
| **IFNg_LatencyAg** |  |  |  |  |  |  |  |  |  | 0.651 |
| **IFNg_TB_Ag** |  |  |  |  |  | 0.547 | 0.363 |  | 0.610 | 0.691 |
| **IL13_TB_Ag** |  |  |  |  |  | 0.259 | 0.454 | 0.117 |  |  |
| **IL5_TB_Ag** |  |  |  |  |  |  | 0.741 |  |  |  |
| **IP10_TB_Ag** |  |  |  |  |  |  |  | 0.231 |  |  |

**(C) Positive predictive value**

|  | **IL2_Latency Ag** | **IL2_TB_ Ag** | **IFNg_Latency Ag** | **IFNg_TB_ Ag** | **IL13_TB_ Ag** | **IL5_TB_ Ag** | **IP10_TB_ Ag** | **TNFa_TB_ Ag** | **CD4_T cell** | **CD8_T cell** |
| --- | --- | --- | --- | --- | --- | --- | --- | --- | --- | --- |
| **IL2_LatencyAg** |  | 0.653 | 0.641 |  |  |  |  |  |  |  |
| **IL2_TB_Ag** |  |  |  | 0.516 | 0.802 | 0.004 | 0.631 | 0.533 |  |  |
| **IFNg_LatencyAg** |  |  |  |  |  |  |  |  |  | 0.670 |
| **IFNg_TB_Ag** |  |  |  |  |  | 0.819 | 0.662 |  | 0.535 | 0.721 |
| **IL13_TB_Ag** |  |  |  |  |  | 0.426 | 0.471 | 0.302 |  |  |
| **IL5_TB_Ag** |  |  |  |  |  |  | 0.663 |  |  |  |
| **IP10_TB_Ag** |  |  |  |  |  |  |  | 0.589 |  |  |

**(D) Negative predictive value**

|  | **IL2_Latency Ag** | **IL2_TB_ Ag** | **IFNg_Latency Ag** | **IFNg_TB_ Ag** | **IL13_TB_ Ag** | **IL5_TB_ Ag** | **IP10_TB_ Ag** | **TNFa_TB_ Ag** | **CD4_T cell** | **CD8_T cell** |
| --- | --- | --- | --- | --- | --- | --- | --- | --- | --- | --- |
| **IL2_LatencyAg** |  | 0.031 | 0.022 |  |  |  |  |  |  |  |
| **IL2_TB_Ag** |  |  |  | 0.347 | 0.571 | 0.557 | 0.688 | 0.432 |  |  |
| **IFNg_LatencyAg** |  |  |  |  |  |  |  |  |  | 0.035 |
| **IFNg_TB_Ag** |  |  |  |  |  | 0.609 | 0.446 |  | 0.048 | 0.012 |
| **IL13_TB_Ag** |  |  |  |  |  | 0.987 | 0.033 | 0.935 |  |  |
| **IL5_TB_Ag** |  |  |  |  |  |  | 0.524 |  |  |  |
| **IP10_TB_Ag** |  |  |  |  |  |  |  | 0.964 |  |  |
